# Integrated omics study delineates the dynamics of lipid droplets in *Rhodococcus opacus* PD630

**DOI:** 10.1093/nar/gkt932

**Published:** 2013-10-21

**Authors:** Yong Chen, Yunfeng Ding, Li Yang, Jinhai Yu, Guiming Liu, Xumin Wang, Shuyan Zhang, Dan Yu, Lai Song, Hangxiao Zhang, Congyan Zhang, Linhe Huo, Chaoxing Huo, Yang Wang, Yalan Du, Huina Zhang, Peng Zhang, Huimin Na, Shimeng Xu, Yaxin Zhu, Zhensheng Xie, Tong He, Yue Zhang, Guoliang Wang, Zhonghua Fan, Fuquan Yang, Honglei Liu, Xiaowo Wang, Xuegong Zhang, Michael Q. Zhang, Yanda Li, Alexander Steinbüchel, Toyoshi Fujimoto, Simon Cichello, Jun Yu, Pingsheng Liu

**Affiliations:** ^1^National Laboratory of Macromolecules, Institute of Biophysics, Chinese Academy of Sciences, Beijing 100101, China, ^2^MOE Key Laboratory of Bioinformatics and Bioinformatics Division, Center for Synthetic and Systems Biology, TNLIST/Department of Automation, Tsinghua University, Beijing 100084, China, ^3^University of the Chinese Academy of Sciences, Beijing 100049, China, ^4^Key Laboratory of Genome Sciences and Information, Beijing Institute of Genomics, Chinese Academy of Sciences, Beijing 100101, China, ^5^Department of Histology and Embryology, University of South China, Hengyang Hunan Province 421001, China, ^6^State Key Laboratory of Microbial Resources, Institute of Microbiology, Chinese Academy of Sciences, Beijing 100101, China, ^7^School of Applied Mathematics, Central University of Finance and Economics, Beijing 102206, China, ^8^Department of Molecular and Cell Biology, Center for Systems Biology, The University of Texas at Dallas, Dallas, TX 75083-0688, USA, ^9^Institut für Molekulare Mikrobiologie und Biotechnologie, Westfälische Wilhelms-Universität, Corrensstrasse 3, D-48149 Münster, Germany, ^10^Environmental Sciences Department, King Abdulaziz University, Jeddah 21589, Saudi Arabia, ^11^Department of Anatomy and Molecular Cell Biology, Nagoya University Graduate School of Medicine, 65 Tsurumai-cho, Showa, Nagoya 466-8550, Japan and ^12^School of Life Sciences, La Trobe University, Melbourne, Victoria, 3086, Australia

## Abstract

*Rhodococcus opacus* strain PD630 (*R. opacus* PD630), is an oleaginous bacterium, and also is one of few prokaryotic organisms that contain lipid droplets (LDs). LD is an important organelle for lipid storage but also intercellular communication regarding energy metabolism, and yet is a poorly understood cellular organelle. To understand the dynamics of LD using a simple model organism, we conducted a series of comprehensive omics studies of *R. opacus* PD630 including complete genome, transcriptome and proteome analysis. The genome of *R. opacus* PD630 encodes 8947 genes that are significantly enriched in the lipid transport, synthesis and metabolic, indicating a super ability of carbon source biosynthesis and catabolism. The comparative transcriptome analysis from three culture conditions revealed the landscape of gene-altered expressions responsible for lipid accumulation. The LD proteomes further identified the proteins that mediate lipid synthesis, storage and other biological functions. Integrating these three omics uncovered 177 proteins that may be involved in lipid metabolism and LD dynamics. A LD structure-like protein LPD06283 was further verified to affect the LD morphology. Our omics studies provide not only a first integrated omics study of prokaryotic LD organelle, but also a systematic platform for facilitating further prokaryotic LD research and biofuel development.

## INTRODUCTION

Lipid droplets (LDs) are cellular organelles widely found in fungal, plant, animal and human cells (1–3). They are encapsulated by a phospholipid monolayer and are compositionally different from other membrane structures (4). They differ in that their primary role is lipid storage, but may also be pivotal in cellular communication with organelles such as the mitochondria to regulate energy metabolism and substrate utilization. LD is an important organelle related to human metabolic diseases and biofuel productions. For example, LD dysfunction is one of the main causes of metabolic disorders such as obesity, insulin resistance, type 2 diabetes, and cardiovascular diseases (5–9). In biofuel studies, triacylglycerol (TAG) in LD of green algae has been investigated and developed for high oil yields by using targeted metabolic engineering (10–12), making it a biological candidate for biofuel production.

Delineating the molecular mechanisms of LD dynamics is essential to understand its formation, functions, synthetic engineering and further biofuel applications. Since *perilipin*, the first protein of perilipin family (PLIN), was identified in 1991 (13), numerous proteins have been revealed to be related to LD functions and dynamics (3,14). LD may also be involved in multiple important cellular processes such as intermembrane lipid traffic (15), lipid storage (16), lipolysis (17), signaling, temporal protein storage (18) and protein degradation (19). LD is reported functionally interacted with many other organelles such as the mitochondria (20), endoplasmic reticulum (21,22), endosome (23) and peroxisome (24). Despite the functional importance of LDs, systematic understanding of the organelle’s biogenesis and dynamics remains elusive. In contrast to eukaryotes that have multiple organelles, LD is the only membranous organelle found in a number of bacterial strains that can be used as ideal model organisms for LD research. Among them, *Rhodococcus opacus* PD630 has the ability to accumulate large amounts of TAG in the LD (25).

The importance of *Rhodococcus opacus* strain PD630 (*R. opacus* PD630) as a model system is also exemplified by its powerful ability to convert carbon sources into lipids. Interestingly, the TAG storage in *R. opacus* PD630 accounts for up to 87% of the cellular dry weight (26), and thus has higher lipid storage capacity when compared with other oleaginous organisms (26,27). Early studies reported that *R. opacus* PD630 has 10 diacylglycerol acyltransferases (DGAT) that assimilate cellular fatty acids into TAG (13,28). Holder *et al*. reported a partial genome, and also performed a comparative genomic study with a lipid mass analysis (29), which identified 16 DGAT and 261 genes that are directly involved in 20 TAG cycle reactions. These previous studies suggest that TAG biosynthesis from carbon sources is a pronounced characteristic of *R. opacus* PD630. Therefore, to facilitate the application of *R. opacus* PD630 LD production for biofuel development, a complete genome of the organism and integrated analysis of its transcriptome, a proteome of its lipid synthesis, storage and metabolism are essential.

We performed multi-omic studies and present herein the complete genome sequence, a comparative transcriptome and a comparative LD proteome of *R. opacus* PD630. After integrating the collected data, a number of protein families involved in LD dynamics were identified including lipid synthesis, LD structure-like proteins, dynamin-like and SNARE-like proteins. A structure-like protein LPD06283 was verified by its LD location and its effect on LD size. Together, these omics are useful tools to investigate the mechanisms of LD dynamics that will enhance our understanding of the lipid storage of LD in biofuel development.

## MATERIALS AND METHODS

### DNA extraction and genome sequencing and assembly

Cells of *R. opacus* PD630 (30) were obtained from Dr Steinbüchel’s lab at the University of Münster. Cells were cultured aerobically in 100 ml of nutrient broth (NB) at 30°C to postlogarithmic phase, and then the DNA was extracted. The complete nucleotide sequence was obtained using a combination of paired-end/mate-pair Illumina sequencing, and 454 sequencing. The sequence gaps were completed by direct sequencing of polymerase chain reaction (PCR)-amplified fragments. For 454 pyrosequencing, genomic DNA was sheared up by nebulization into random fragments of 500–800 bp for the construction of a dispersed library, which was then clonally amplified and sequenced on a 454 Genome Sequencer. For Illumina sequencing, genomic DNA was processed to construct paired-end libraries with size spans of 300 bp, and also mate-pair libraries with size spans of 3 kb using an Illumina Genomic DNA Sample Prep kit.

The total number of 454 reads obtained was 861 751, giving a 36-fold coverage, while the total number of paired-end and mate-pair library reads was 40 110 584, giving a 445-fold coverage. We used two assembly programs and combined the primary contigs and paired-end data to build scaffolds in successive assemblies. Four hundred fifty-four sequences were assembled using the Roche GS assembler, Newbler (version 2.5), with default parameters. The primary contigs were then scaffolded with Illumina mate-pair reads using SSPACE-premium (version 2.1) (31). To close the gaps among scaffolds, read pairs that were uniquely mapped to the contig tails were extracted for manual assembly. Primers were designed for the remaining gaps and PCR walking was used to finish the whole genome. Illumina reads (300 bp) were mapped to this assembled whole genome sequence to identify potential single miss-called nucleotides using the Bowtie method (32).

### Genome analysis and annotation

Gene models were predicted independently using GLIMMER (33) and GeneMark (34). The predicted open reading frames (ORFs) were further evaluated and adjusted using RBSfinder (35). The translated sequences of the predicted protein-coding genes were searched against UniProt (36) and InterPro (37). The function of enzymes was assigned using EFICAz2 (38) and searched against the KEGG database (39). We used the COG classification scheme (40) to further classify gene functions. Two-tailed Fisher exact test was used to compare the distributions of COG categories between two species. For each COG category, a 2 × 2 contingency table was constructed by recording the numbers of genes included or not included. Putative tRNAs were identified using tRNAscan-SE (41) and TFAM 1.0 (42). rRNAs were detected by RNAMMER (43) and confirmed against known rRNAs using BLASTN. Transposons and repeat elements were identified using ISfinder (44) and searched against Repbase (45). Protein domains were predicted using the Pfam (46) and NCBI CDD (47) databases. Horizontally transferred genes (HTGs) were predicted using the WN method (48). For protein sequence analysis, multiple alignments were generated with CLUSTALX (49), and phylogenetic analysis was performed with MEGA4.0 (50). The operons of PD630 were predicted by using a statistic operon prediction method (51). All PD630 proteins were compared with the proteins of *R. opacus* B4 (*R. opacus* B4), *Rhodococcus jostii* RHA1 (*R. jostii* RHA1) (52) and an earlier reported partial genome of PD630 (29) that were downloaded from NCBI database (NCBI release data of March 2012), by using BLASTP (53) with a cutoff value of 1.0E-3.

### RNA extraction, sequencing and transcriptome analysis

RNA was extracted from *R. opacus* PD630 under the three culture conditions, NB, MSM3 and MSM24, by using Trizol Reagent (Invitrogen, Carlsbad, CA, USA) following the standard protocol except that after isopropanol treatment of the sample was incubated at −20°C overnight. Further purification and DNase treatment was conducted with RNAprep pure cell/bacteria and RNAclean Kits (TIANGEN, Beijing, China) according to the manufacturer’s instructions. *Rhodococcus opacus* PD630 rRNA was depleted using a RiboMinus Eukaryote Kit (Invitrogen, Carlsbad, CA, USA). After RNA amplification, libraries were constructed for sequencing by using a SOLiD system (Applied Biosystems Inc.) according to the manufacturer's specifications.

RNA-Seq reads from each mRNA sample were mapped against our assembled genome by using Bowtie with the ‘best’ strata option (32). Totals of 39 922 375, 62 306 706 and 51 153 776 reads from the NB and MSM3 and MSM24 samples, respectively, were mapped with less than two mismatches. To analyze differential expression, fragments per kilobase of transcript per million mapped reads values (FPKM) were calculated using Cufflinks (54). The fold change between conditions 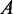 and 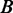 is calculated as 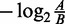.

### Quantitative real-time PCR

Total RNA from cultured *R. opacus* PD630 was isolated using Trizol Reagent (Invitrogen) and purified using TIANGEN RNAclean Kit (TIANGEN) according to the manufacturer’s instructions. For quantitative real-time PCR (qPCR) analysis, RNA was reverse transcribed using the M-MLV Resverse Transcriptase Kit (Promega) and further used in qPCR reactions containing SYBR green fluorescent dye (ABI). Relative expression of mRNA was determined after normalization with 16S levels using the DD-Ct method, comparing MSM3, MSM24 with NB, respectively. qPCR was performed using an ABI StepOne PLUS PCR machine.

### LD purification

LD was isolated according to the method described by Ding *et al.* (55). Forty milliliters of *R. opacus* PD630 cells were centrifuged in NB, and then transferred into 400 ml of mineral salt medium (MSM) and cultured for 24 h for TAG accumulation. MSM contains a high carbon source (10 g/l) but low nitrogen source (0.5 g/l), and primarily used to induce a stress state in the culture medium for TAG accumulation. Cells were collected by centrifugation at 5000*g* for 10 min and washed twice with 30 ml of phosphate buffered saline (PBS) each time. After incubating in 30 ml of buffer A (25 mM tricine, 250 mM sucrose, pH 7.8) on ice for 20 min, cells were homogenized by passing through a French Pressure Cell four times at 100 MPa, and 4°C. The cell homogenate was centrifuged in a 50-ml tube at 6000 *g* for 10 min to remove cell debris and unbroken cells. The postnuclear supernatant fraction (10 ml) overlaid with 2 ml of buffer B (20 mM HEPES, 100 mM KCl, 2 mM MgCl_2_, pH 7.4) was centrifuged at 38 000 rpm for 1 h at 4°C (Beckman SW40). The white band containing LDs at the top of the gradient was collected using a 200-μl pipette tip and transferred to a 1.5-ml Eppendorf tube. LDs were washed three times with 200 μl of Buffer B each time. One milliliter of chloroform:acetone (1:1, v/v) was added to each sample to dissolve lipids and precipitate LD proteins. The sample was mixed thoroughly by vortexing and then centrifuged at 20 000 *g* for 10 min (Eppendorf centrifuge 5417R). The pellet containing LD proteins was resolved with 50 μl of 2× sodium dodecyl sulphate (SDS) sample buffer and denatured at 95°C for 5 min. The sample was stored at −20°C until required.

### Mass spectrometry (MS) analysis

The bands of interest from the NB and MSM24 samples were cut from SDS-polyacrylamide gel electrophoresis (SDS-PAGE) gels. Samples were loaded onto a C18 trap column with an auto-sampler and then eluted onto a C18 column (100 mm × 100 μm) packed with Sunchrom packing material (SP-120-3-ODS-A, 3 μm) for nano-LC-ESI-LTQ MS/MS analysis. The linear trap quadrupole (LTQ) mass spectrometer was operated in data-dependent mode with the initial MS scan ranging from 400–2000 Da. All the MS/MS data were searched against our assembled and annotated genome sequence by the SEQUEST program (Thermo, USA). Bio-Works search parameters were set up as enzyme, trypsin, precursor ion mass tolerance, 2.0 Da; and fragment ion mass tolerance, 1.0 Da. The variable modification was set to oxidation of methionine (Met + 15.99 Da) and the fixed modification to carboxyamidomethylation of cysteine (Cys + 57.02 Da). Results were filtered with Xcorr (charge values) of Xcorr (+ 1) > 1.90, Xcorr (+ 2) > 2.50 and Xcorr (+ 3) > 3.75, where Xcorr is the cross-correlation score of a candidate peptide against a search database. The MS/MS data were then converted and deposited at PRIDE database (56).

### Construction of LPD06283 deletion mutant

A deletion mutant of structural protein LPD06283 was constructed by using homologous recombination. Colloidal Blue staining was used to verify the absence of the LPD06283 protein bands. Different phenotypes between the LPD06283 deletion mutant strain and the wild type were observed by EM. The upstream and downstream sequences of the target gene were cloned by PCR using primers a/b and c/d, respectively, and using the wild type *R. opacus* PD630 genome as a template, generating fragments AB and CD. Fragments AB and CD were ligated together, sequenced and then cloned into a pK18mobsacB plasmid. Plasmid pK18mobsacB was kindly provided by Ping Xu from Shanghai Jiao Tong University. The pK18mobsacB fusion plasmids were transformed into *R. opacus* PD630 by electronic transformation. Positive mutants were selected with a positive screen using a kanamycin cassette and a negative screen using a sacB cassette. Primers a and d were used to confirm that the final selected cells were positive mutants. Primers f and r were used for further PCR validation.

All primer sequences were as follows:

LPD06283-a: CGGAATTCTGAGGAGTTCACTGA TGGTGGCG

LPD06283-b: CGGGATCCTGCGTGTCGACCTCG TAGGATGGG

LPD06283-c: CGGGATCCCGGCTTTCTCCTGTTC AACGGTGG

LPD06283-d: CGAAGCTTAAGAAGATCGAGCTG CAGGTGGGG

LPD06283-f: CAGGATCCACTGACCAGAAGACC ATCGACAGCGT

LPD06283-r: CAGGATCCAGCCTTCTTGGCCGGA GCAGCCTT

### Thin layer chromatography and western blotting

For thin layer chromatography (TLC), neutral lipids were extracted twice from purified LD and bacterial samples using chloroform:acetone (1:1, v/v) and chloroform: methanol:medium (1:1:1, v/v/v), respectively. The organic phases were collected and air dried with nitrogen gas of a high purity. Total lipids were dissolved in 100 µl of chloroform for TLC analysis by using Whatman Purasil^TM^ 60FÅ silica gel plates (Merck, Germany). Neutral lipids were developed using the solvent system hexane:diethyl ether:acetic acid (80:20:1, v/v/v) and phospholipids in chloroform:methanol:acetic acid:H_2_O (75:13:9:3, v/v/v/v). TLC plates were visualized using iodine vapor.

For western blotting, proteins were separated by SDS-PAGE and transferred to a polyvinyl difluoride (PVDF) membrane, followed by blotting with the antibodies indicated and detection using an ECL system. We selected 20 LD proteins based on the proteome analysis for antibody production. Two rabbits were immunized with two synthetic peptides per protein.

### Transmission electron microscopy and confocal microscopy

Bacterial cells were examined by transmission electron microscopy (TEM), including positive staining and ultrathin sectioning methods. For positive staining, cells were loaded onto carbon-coated copper grids and subsequently stained using 2% (w/v) phosphotungstic acid for 2 min. The grid was then washed with deionized water thrice before viewing using a FEI Tecnai 20 (FEI Co., Netherlands) electron microscope. For ultrathin sectioning, cells were prefixed in 2.5% (w/v) glutaraldehyde in PBS (pH 7.4) overnight at 4°C and postfixed in 2% (w/v) potassium permanganate for 5 min at room temperature. The sample was then dehydrated in ascending concentrations of ethanol at room temperature and embedded in Spurr’s resin. Sections with a thickness of 70 nm were cut with a Leica EM UC6 Ultramicrotome (Leica Germany), then stained with 2% (w/v) uranyl acetate for 15 min and lead citrate for 5 min at room temperature before visualization.

For confocal microscopy, PD630 cells were washed twice with PBS and then mounted onto coverslips pretreated with collagen prepared from rat tail. Samples were dried for 30 min before washing with 1 ml of PBS, and then incubated for 30 min in a 1:500 solution of LipidTOX Red in darkness at room temperature. Samples were mounted onto glass slides with Mowiol mounting media and analyzed by confocal microscopy (Olympus FV1000).

### Data access

Genome assemblies, together with predicted gene models and annotation, were deposited at GenBank under the project accession number PRJNA178618. The accession numbers of chromosome and plasmids are CP003949 (chromosome), CP003950 (plasmid-1), CP003951 (plasmid-2), CP003952 (plasmid-3), CP003953 (plasmid-4), CP003954 (plasmid-5), CP003955 (plasmid-6), CP003956 (plasmid-7), CP003957 (plasmid-8), CP003958 (plasmid-9). The expression data sets used in this study are available at the NCBI Gene Expression Omnibus (GEO) (http://www.ncbi.nlm.nih.gov/geo/), under accession number GSE42381.

## RESULTS

### *Rhodococcus opacus* PD630 genome exhibits a super ability of biosynthesis and catabolism

**Figure 1.**
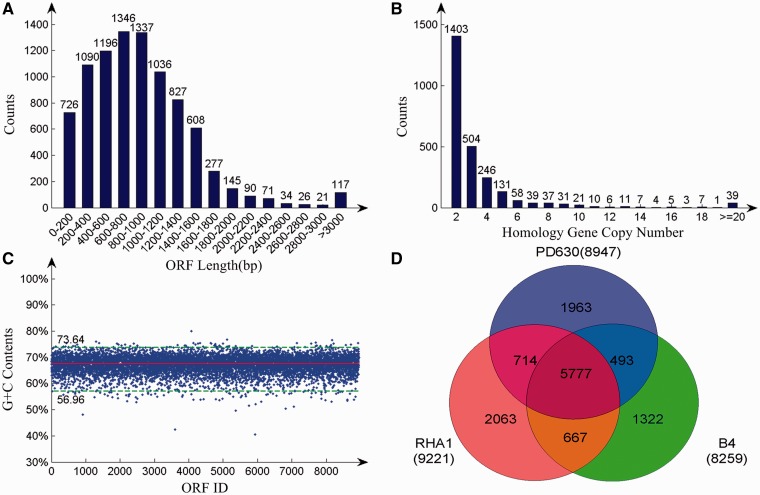
Basic statistic information of *R. opacus* PD630 genome. (**A**) Length distribution of predicted *R. opacus* PD630 ORFs. The average length of all genes is 928 bp, with most genes ranging from 200 to 1600 bp. The number of genes in each category is noted above each bar. (**B**) Distribution of the copy numbers of homologous genes among all 8947 genes. Homologous genes were detected using BLASTP with an e-value cutoff of 1.0E-20 and identity of 50%. The number of genes in each category is noted above each bar. (**C**) The average C + G content of all genes was 67.47% (solid red line). One percent of the genes fell outside the upper and lower green-dotted lines and had markedly higher or lower G + C contents. (**D**) Venn diagram comparing the whole genomes of *R. opacus* PD630, *R. jostii* RHA1 and *R. opacus* B4. All proteins in *R. opacus* PD630 were compared with those in *R. jostii* RHA1 and *R. opacus* B4 using BLASTP with an e-value cutoff of 1.0E-20 and an identity of 50%.

We used a combined dispersed strategy incorporating data generated using Roche/454 and Illumina sequencing technologies, and assembled the genome and associated plasmids of *R. opacus* PD630. The complete genome consists of a circular chromosome of 8 376 954 bp in length and nine plasmids, which in combined total are 9 169 032 bp (Supplementary Table S1). The genome encodes 8947 protein-coding genes, 51 tRNA and 12 rRNAs genes (Supplementary Tables S2 and S3). The mean size of predicted ORFs is 928 bp, with 80.28% of the ORF between 201 and 1500 bp in length (Figure 1A). Further, a few insertion sequences were predicted using the ISfinder (44) (Supplementary Table S4), but only occupy 0.38% of the genome. A large number of genes (i.e. 2563) are duplicated (Figure 1B). *Rhodococcus opacus* PD630 has a high genomic G + C (guanine–cytosine content) content (67.47%), with 176 genes having either a much higher or lower G + C contents than the genome on average (Figure 1C). Protein functions were manually assigned based on Interpro (37), UniProt searches (36) and COG functional classification system (40). Among the 22 COG subclasses, lipid transport and metabolism were the largest category, occupying 850 genes.

The genomes of two other species in the *Rhodococcus* genus, namely *R. opacus* B4 (*R. opacus* B4) and *R. jostii* RHA1 (*R. jostii* RHA1) (52) have also been sequenced. Although they are closely related to *R. opacus* PD630 (Figure 1D), comparative analyses reveals that *R. opacus* PD630 may have superior lipid metabolism. For example, *R. opacus* PD630 has 850 COG categories E genes (amino acid transport and metabolism), 556 of the P genes (inorganic ion transport and metabolism) and 580 of the G genes (carbohydrate transport and metabolism), which is significantly more than *R. jostii* RHA1, which has 595, 329 and 434 genes, respectively, and *R. opacus* B4, which has 575, 328 and 412 genes, respectively, in the above mentioned categories (all *P* < 1E-04, two-tailed Fisher exact test, Supplementary Table S5). Enzymes were annotated to metabolic reactions using EFICAz2 (38) and the KEGG database (39). In summation, 3200 enzymes are predicted for 2727 metabolic reactions that are markedly more than predicted reactions of *R. jostii* RHA1, *R. opacus* B4 (39) and earlier reported 2017 metabolic reactions of PD630 partial genome (29).

As *R. opacus* PD630 is able to grow on a variety of substrates, and environments (26,29,57), we searched for HTGs that might confer selective evolutionary advantages in different environments. A total of 532 genes (Supplementary Table S2) were predicted as HTG by using the WN method (48). Of the 612 genes implicated in energy production and conversion, and the 711 genes involved in lipid transport and metabolism, 73 and 77, respectively, were predicted to be HTG, providing significant evolutionary contributions to lipid synthesis and lipid metabolism in *R. opacus* PD630 (*P*-values: 2.26E-07 and 3.05E-06, two-tailed Fisher exact test).

### Global gene expressions under lipid accumulation

**Figure 2.**
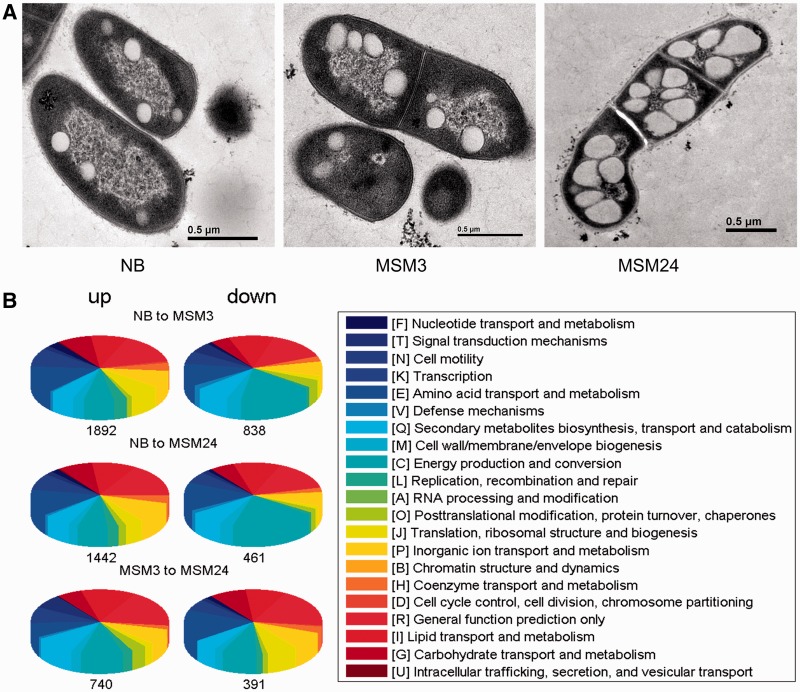
Whole-genome differential expression analysis. (**A**) EM images (ultra-thin sections) of *R. opacus* PD630-WT cultured in MSM for 3 h (MSM3), or 24 h (MSM24), or grown in NB for 48 h (NB). Bar = 0.5 μm. (**B**) Relative abundance of different categories of differentially expressed genes in *R. opacus* PD630 are shown under the three culture conditions. Pie charts on the left represent upregulated genes, while those on the right represent downregulated genes. The total number of genes accounted is given below each pie chart. Colors correspond to categories in the COG database.

To confirm that *R. opacus* PD630 cells include LDs and also display their dynamics in different cultures, cells were first cultured in NB, and subsequently transferred to a MSM for 3 h (MSM3) and 24 h (MSM24), respectively. Since MSM is a low nitrogen/carbon ratio medium that is used as a stressful culture for TAG accumulation (27,28,58), the systematically comparative analysis of differential gene expressions under NB and MSM cultures will be helpful to reveal how proteins and biological pathways respond to TAG accumulation and LD dynamics. Firstly, LipidTOX staining showed that the *R. opacus* PD630 cells grown in MSM cultures contain much larger LDs than those grown in NB culture (Supplementary Figure S1A). This observation is in agreement with results from the TLC that TAG is accumulated to a greater amount in MSM24 than NB, but DAG is much accumulated in lesser quantities (Supplementary Figure S1B). Further, we imaged cells by TEM to better visualize phenotypic differences in LDs. Interestingly, LDs derived from the MSM24 treatment were much larger in diameter than those cultured in NB (Figure 2A). Moreover, *R. opacus* PD630 contains many electron-transparent structures that occupy most of the inner area of the cell, with the TAG content increasing from the NB to MSM24 culture (Supplementary Figure S2). These measurements confirm that *R. opacus* PD630 contains LDs and accumulate large amounts of TAG under MSM culture conditions.

To investigate the proteins that are related to dynamics of *R. opacus* PD630 under different culture conditions, we sequenced and compared whole-genome transcriptomes of these three cultures NB, MSM3 and MSM24. The quality of our transcriptomes was confirmed by measuring expressions of 13 randomly selected genes by using qPCR. Among the 13 genes, 9 genes (LPD05955, LPD07778, LPD02638, LPD05411, LPD04190, LPD04189, LPD05410, LPD02774, LPD06334) presented similar trends in NB to MSM3 and MSM24, and only four genes (LPD02936, LPD05356, LPD02250, LPD07707) had little ratio differences (Supplementary Figure S3 and Supplementary Table S6 for detailed expression values). The qPCR result confirmed the transcriptomic data is reliable. We then performed a systematic analysis of the genome-wide expression dynamics under three cultures NB, MSM3 and MSM24. Most genes were either expressed under all three conditions (6759 genes) or under at least one condition (7770 genes), but there were 1177 genes that were not expressed under any of these conditions. When cells were changed from NB to MSM condition, a drastic response to environmental change was observed even after 24 h. We observed a marked response 3 h after cells were transferred from NB to MSM; with 56.99% of the genes upregulated and 30.32% downregulated, and 21.15% being upregulated >2-fold and 9.36% being downregulated >2-fold (Supplementary Figure S4). The functional enrichment analysis was performed for differentially expressed genes (≥2-fold change; Figure 2B, Supplementary Table S7). Genes in category J (translation, ribosomal structure and biogenesis) were significantly upregulated (*P*-value: 5.05E-20, two-tailed Fisher exact test), while genes in category K (transcription), G (carbohydrate transport and metabolism) and C (energy production and conversion) were downregulated (*P*-values of 6.0E-07, 8.08E-06 and 4.56E-05, respectively, two-tailed Fisher exact test). The increased expression of ribosomal proteins is consistent with the number of genes (56.99%) upregulated in the MSM3 treatment. The large upregulated protein proportions and their functional enriched groups exhibited a dynamical landscape of proteins and pathways responsible for lipid synthesis and storage in PD630.

### Enzymes involved in TAG biosynthesis and metabolism

**Figure 3.**
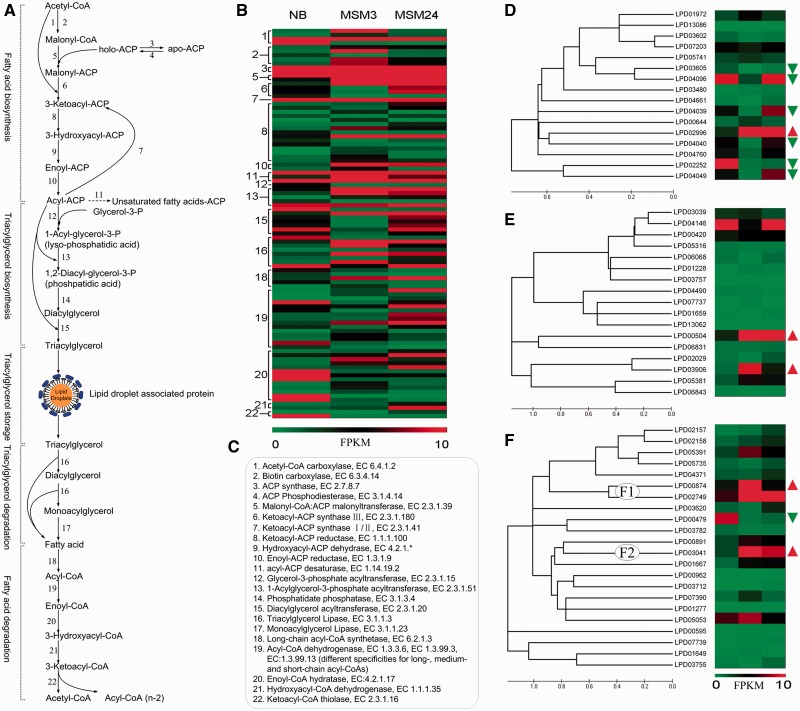
Expression and phylogenetic analysis of gene families involved in TAG biosynthesis and degradation. (**A**) TAG biosynthesis, storage and degradation pathways are divided into five biochemical stages and 22 reactions. (**B**) Heatmap of highly expressed (≥10 FPKM) or dramatically differentially expressed enzymes (≥2-fold change) in each reaction. (**C**) EC numbers of enzymes involved in (A). (**D**) Phylogenetic tree and heatmap of 16 predicted TAG synthases. (**E**) Phylogenetic tree and heatmap of 17 predicted TAG lipases. (**F**) Phylogenetic tree and heatmap of 22 predicted esterases. Genes whose expression increased from NB to MSM3 (≥2-fold change) are marked by red triangles, and those which decreased (≤2-fold change) are marked by green triangles in D, E and F. Two examples of gene clusters that have similar sequences and expression patterns are noted as F1 and F2.

The accumulation of TAG in *R. opacus* PD630 is a dynamic balance between lipid synthesis and degradation. Here we systematically analyzed the potential proteins involved in the TAG biosynthesis and metabolic pathway. The pathway consists of 22 reactions, involving 457 candidate enzymes (Supplementary Table S8). These reactions are further classified into five stages: initially fatty acid biosynthesis, TAG biosynthesis, TAG storage, TAG degradation and fatty acid degradation (Figure 3A and C). In these enzyme families, the largest one is 123 3-oxoacyl-(acyl-carrier-protein) reductase (EC:1.1.1.100), exhibiting a marked preference for acyl-carrier-protein derivatives over CoA derivatives as substrates. Hundred acyl-CoA dehydrogenase (EC:1.3.3.6/EC:1.3.99.3/EC:1.3.99.13) performed different specificities for long, medium and short chains. Other large gene families include 16 diacyglycerol O-acyltransferase (DGATs, EC:2.3.1.20), 45 lipase/esterase (EC:3.1.1.3), 40 long-chain fatty acid CoA ligase (EC:6.1.2.3) and 53 enoyl-CoA hydratase (EC:4.2.1.17). Among these reactions, many enzymes were highly expressed or differentially expressed by >2-fold change (Figure 3B), indicating that a broad spectrum of fatty acids were differentially synthesized and catabolized in *R. opacus* PD630 when cultures changed.

Earlier studies revealed that *R. opacus* PD630 can synthesize many neutral lipids, but the precise enzymes involved for different lipids have rarely been reported (13,28–30). Thus, identifying key lipid enzymes and suppressing glycogen synthesis appears important to ensure maximal TAG yields. In our study, 16 DGATs were predicted as possessing lipid synthases activity and these could be further divided into several subclusters using phylogenetic analysis (Figure 3D). The expression of genes in each subcluster was similar under three conditions, indicating that those genes of subclusters may be involved in different lipid synthesis. In particular, the gene LPD02996 was significantly upregulated >2-fold change in MSM3 compared with NB culture. Three genes, LPD01972, LPD05741 and LPD00644, were also slightly upregulated with fold change of 1.39, 0.41 and 1.34, respectively. Since TAG accumulates to high levels in MSM cultures, the increased expression of these four genes suggests that they may be involved in TAG synthesis. LPD05741, also named *artf2*, had been verified to be involved in TAG biosynthesis and accumulation in PD630 (59). The elevation of LPD05741 is consistent with this study. Furthermore, the significant upregulation of LPD02996 also suggests it may be another potential protein related to TAG biosynthesis. Six genes LPD03605, LPD04096, LPD04039, LPD04040, LPD02252 and LPD04049 were significantly downregulated >2-fold change, indicating that these six genes may be involved in other lipid synthesis, or TAG degradation but blocked in MSM culture to potentiate greater TAG yield. A total of 45 genes coding for putative lipase/esterase proteins (17 lipases, 22 esterases and 6 others) were predicted to be involved in neutral lipid degradation. Of the 17 lipases, the expression of LPD00504 and LPD03906 were significantly elevated in MSM3 compared with NB (≥2 fold change), while LPD03039, LPD00420 and LPD05381 were slightly increased with fold change of 0.2, 0.44 and 1.84, respectively. A gene LPD04146 was decreased with a fold change of 1.68 in MSM3 compared with NB (Figure 3E). Of the 22 esterases, LPD00874 and LPD03041 were dramatically upregulated (≥2-fold change), while LPD05391 LPD02749, LPD00891, LPD01667, LPD07390 and LPD05053 were slightly upregulated with fold change of 1.58, 1.24, 1.46, 1.86, 1.55 and 0.31, respectively. Gene LPD00479 was significantly downregulated in MSM3 compared with NB culture (≤2-fold change, Figure 3F). These proteins can be classified into some subclusters with similar expression patterns in each cluster by phylogenetic analysis. For example, LPD00874 and LPD02749 are both increased in MSM3 and then decreased in MSM24 (Figure 3F-F1). A similar tendency is also observed among LPD00891, LPD03041 and LPD01667 (Figure 3F-F2). These results presented a precise prediction that those differentially expressed lipases/esterases may be involved in TAG synthesis and degradation.

### Identifying proteins associated to prokaryotic LD

**Figure 4.**
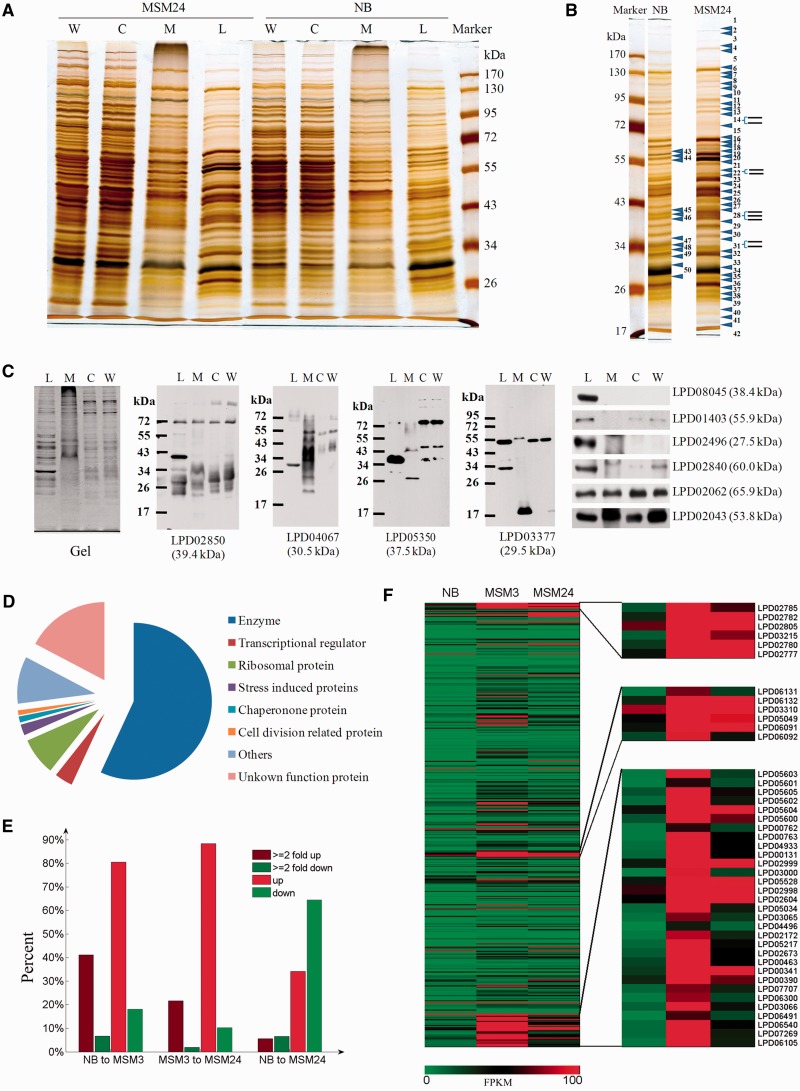
Functional and expressional analysis of LD proteins. (**A**) *Rhodococcus opacus* PD630 proteins from whole-cell lysates (W), cytosol (C), membranes (M) and purified LDs (L) were separated by 10% SDS-PAGE, followed by silver staining. (**B**) LC-MS band analysis of LD-associated proteins. Arrows indicate the positions at which the gel was sliced. Forty-two bands (1–42) from the MSM24 sample, and eight bands (43–50) from the NB sample corresponding to the major bands in the MSM24 sample were analyzed. (**C**) Western blotting of LD-associated proteins. The same amount of protein from LDs, membranes, the cytosol and whole-cell lysates were separated by 10% SDS-PAGE and blotted with the antibodies indicated. Western blotting using anti-LPD02850, anti-LPD04067, anti-LPD05350, anti-LPD03377, anti-LPD08045, anti-LPD01403, anti-LPD02496, anti-LPD02840, anti-LPD02062 and anti-LPD02043. (**D**) 430 LD-associated proteins were identified in MSM24 and categorized into nine groups based on searches of our *R. opacus* PD630 genome, and the Pfam and NCBI databases. (**E**) Histogram showing the percentage of LD-associated genes with dramatic expression changes in the three conditions. (**F**) Heatmap showing the expression of all 430 genes. ATP synthase (up), RNA polymerases, translation initiation factors and elongation factors (middle) and ribosome proteins (down) are enlarged to the right of the heatmap.

To better understand prokaryotic LD proteins, we isolated LDs (please see ‘Materials and Methods’ section) (Supplementary Figure S5A) and performed proteomic and lipid analyses. Initially, we determined the quality of isolated LDs. TEM imaging of isolated LDs using positive staining displayed few contaminants present from other membranes (Supplementary Figure S5B). The size distribution of LDs is bell shaped, and between 131 and 3168 nm as determined using a Delsa Nano C particle analyzer (Supplementary Figure S5C). Total lipids from isolated LDs and from the total cell membrane were separated by TLC (Supplementary Figure S5D). Results indicated that the main lipid present was TAG (band 2 in Supplementary Figure S5E), and that the total membrane fraction was enriched in phosphatidylethanolamine (band 5 in Supplementary Figure S5F) and also an unknown lipid (band 4 in Supplementary Figure S5F). The protein composition of LDs was distinctly different from that of the total membrane, cytosol and whole-cell lysates (Figure 4A), further verifying the high quality of the isolated LDs.

We then conducted comparative proteomic studies on LDs isolated under different culture conditions, and focused our attention to identify proteins that are related to LD functions and dynamics. LD proteins from bacteria cultured in MSM24 were initially separated by SDS-PAGE. The gel was cut into 42 slices, with 430 proteins identified using proteomic analysis (Figure 4B). Proteins with a high abundance according to the peptide number in the MS data were chosen and respective antibodies produced. Further, Western blotting was performed to verify the association of these proteins with LDs and determine their cellular distribution (Figure 4C). The proteins LPD02850, LPD04067, LPD05350, LPD03377, LPD08045 and LPD02496 were mainly present in the LD fraction, whereas proteins LPD01403 and LPD02840 were present in both the LD and cytosol fractions. Proteins LPD02062 and LPD02043 were ubiquitous distributed within the whole cell.

The 430 proteins identified in LDs were categorized into nine groups, including 231 enzymes, 6 transport proteins, 25 transcription and translation proteins, 31 ribosome proteins, 5 chaperone protein, 8 stress-induced proteins, 5 cell division–related proteins and 110 other unknown functional proteins (Figure 4D and Supplementary Table S9). The 231 enzymes were mainly involved in lipid synthesis and degradation, including 40 transferases, 8 ligases, 37 dehydrogenases, 32 reductases and 29 synthases, which summed up >63% of these enzymes. To further confirm these results, two independent proteomic analyses of PD630 LD proteins under MSM24 condition were performed. Two hundred thirty-eight of these 430 proteins were also identified at least once (Supplementary Table S10). The major LD protein bands that showed marked differences between the NB and MSM24 samples (Figure 4B, bands 43 to 50. Supplementary Table S11 for corresponding bands in two samples) were then subjected to proteomic analysis. Most abundant proteins in these bands were similar in these two samples, but increased markedly in the MSM24 samples compared with NB. Interestingly, we found that 31 ribosomal proteins were all increased by >2-fold change (Supplementary Table S9). Furthermore, six ATP enzymes (Figure 4F, LPD02785, LPD02782, LPD02805, LPD03215, LPD02780 and LPD02777), two RNA polymerases, two translation initiation factors, two elongation factors and 15 transcriptional regulators were also found to be differentially expressed. There were 13 of the 15 transcriptional regulators upregulated in MSM3 compared with NB. Since these expressions of these factors were highly correlated, they may construct as a complete transcription/translation system in LD surface. For summary, the abundant proteins detected in PD630 LD revealed that prokaryotic LD are involved in not only the lipid synthesis and catabolism, but also multiple other important cellular functions.

### Proteins involved in LD dynamics

Integrating transcriptomic and proteomic data is a useful method to reveal the proteins involved in LD dynamics. Transcriptome analysis specifically for LD proteome showed that >50% of 430 proteins were dramatically differentially expressed under the varying NB, MSM3 and MSM24 conditions. Totally, 177 of the 430 genes were significantly upregulated, while 30 genes were downregulated (≥2-fold change or ≤2-fold change) from NB to MSM3 (Figure 4E), suggesting that these proteins play important functions related to LDs when culture conditions changed. Specifically, 99 proteins of the 231 enzymes were increased, suggesting that the lipid synthesis processes are much accelerated. We also identified four dynamin (LPD02043, LPD02044, LPD02062 and LPD02063) and three SNARE-like proteins (LPD02118, LPD02119 and LPD03976) that may be involved in LD dynamics (Supplementary Table S12). Each of the dynamin proteins contains a Dynamin_N domain (PF00350) (also named DLP_2 in NCBI CDD database) (Supplementary Figure S6A). Further, operon LPD02062 and LPD02063 were highly expressed but operon LPD02043 and LPD02044 showed almost no expressions. The three SNARE-like proteins each contain a SNARE_assoc domain (PF09335) (also named PRK10847 in NCBI CDD database) (Supplementary Figure S6B). Moreover, LPD03976, LPD02118 and LPD02119 were expressed under all three conditions and were observed to have a 1.5-fold change from NB to MSM3 culture. Since dynamin and SNARE-like proteins play important roles in LD budding and fusion processes in eukaryotes (60,61), their presence in *R. opacus* PD630 suggests that these proteins may also mediate the LD dynamics in prokaryotic organisms, thus indicating their evolutionary origins. So far, these integrated omics studies systematically revealed multiple factors that are related to prokaryotic LD dynamics, especially the lipid synthesis, storage and LD morphology.

### A structure-like protein affects the morphology of prokaryotic LDs

**Figure 5.**
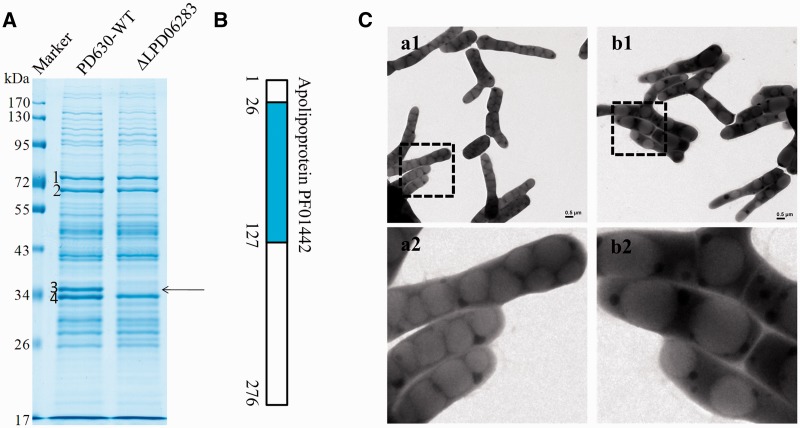
Deletion of LPD06283 results in supersized LDs. (**A**) Gel electrophoresis of LD proteins from *R. opacus* PD630-WT and the LPD06283 deletion mutant stained by colloidal blue. Band 3, the main band, disappeared in the LPD06283 deletion mutant. (**B**) Location of the predicted domain in LPD06283, Apolipoprotein (PF01442). (**C**) a1-a2, EM images of *R. opacus* PD630-WT cultured in MSM for 24 h after growing in NB for 48 h using positive staining methods; b1-b2, EM images of the LPD06283 deletion mutant under the same conditions as *R. opacus* PD630-WT. The lower panels give amplified pictures. Bar = 2 μm.

Among all the proposed proteins that may be involved in LD dynamics, we further determined the protein LPD06283 as a structure-like protein. We observed that it was involved in LD dynamics. The expression of LPD06283 was markedly increased from NB (FPKM 3.77) to MSM3 (FPKM 18.15), achieving a 2.27-fold change. It was revealed as the major protein band (Figure 5A). A fragment of LPD06283 (from 26 to 127 bp) was predicted to be an apolipoprotein domain (PF01442, Figure 5B) by using Pfam databases (46). The average posterior probability that means the degree of confidence in each individual aligned residue is arranged from 65 to 75%, showing a high confidence of this prediction. We deleted it by homologous recombination to investigate its function (Supplementary Figure S7A and B). Compared with the wild type, LDs in the LPD06283 deletion mutant were dramatically larger; however, their numbers were reduced (Figure 5C, Supplementary Figure S8, S9). LPD06283 is sequence similar with the earlier reported proteins Ro02104 in RHA1 (62) and TadA in PD630 (63). These results are consistent with early studies demonstrating that LDs are easily fused in the absence of structural proteins, and also consistent with early studies in eukaryotic cells (62,64-66).

### DISCUSSION

LD is an important organelle in both prokaryote and eukaryote organisms, but little is known regarding its function, dynamics and evolution. *R. opacus* PD630 is a useful model for the investigation of LD, since the LD is the only intracellular membranous organelle. We have comprehensively presented the *R. opacus* PD630 genome, transcriptome and LD proteome, and have systematically investigated the key proteins potentially involved in LD dynamics and functions. Integrating these oimcs data revealed significantly differential expression of 177 LD proteins and further depicted a dynamical landscape of prokaryotic LD in TAG accumulating cultures. These results not only confirmed several early studies of LD proteins, but also indicated many novel candidate proteins for optimizing TAG synthesis and storage.

Comparative proteomics detected an abundance of proteins on the PD630 LD surface, including enzymes, transport protein, cell division related proteins, transcription/translation protein, ribosome proteins and many other unknown proteins. These diverse proteins indicate that prokaryotic LDs, as the first intracellular membrane system, are involved in lipid metabolism and other important cellular functions. Furthermore, this diversity of LD proteins was also observed in earlier reported proteomics from multiple species, such as green algae (67), yeast (68), *Drosophila* (69), *Caenorhabditis elegans* (70), mice (71), indicating a functional evolutionary path from prokaryotes to mammals. For example, many ribosomal proteins were found in the LDs of PD630, yeast, *C. elegans* and Drosophila embryos but absent in the LDs of mice. The number of enzymes presented in LDs is decreased from PD630 to mice. The wide range of functions carried out by prokaryotic LDs and the specialization of mammalian LDs suggest that functional specialization, accompanied by the distribution of other biochemical processes among other organelles, and it is a trend that continues during LD evolution from prokaryotes to eukaryotes.

In both prokaryotic and eukaryotic cells, LDs have the same topological structure that lipids are encapsulated by phospholipid monolayer. From a topological view, LD should be evolutionarily constructed by cells themselves, and rather different from organelles such as the mitochondria and chloroplast that may be original via fusion of prokaryotic cells (72,73). An early model of LD ontogeny in strain PD630 had been proposed that LDs originate in the internal side of the cytoplasmic membrane, where DGAT enzymes are localized (58). However, this model presents difficulty to explain how TAGs are synthesized and topologically encapsulated into LDs in the cytoplasm, as TAGs are not soluble in water. Our discovery of dynamin-like proteins in LDs may suggested that the initialized lipid synthesis may be accelerated at the cell membrane bilayer and disengaged from cell membrane via catalysis of dynamin-like proteins; however, further experiments need to be performed to validate this hypothesis. We believe the complete *R. opacus* PD630 genome, transcriptome and LD proteome presented here provides a starting point not only for unravelling mechanisms of LD dynamics but also for investigating the organelle and eukaryotic evolution.

## SUPPLEMENTARY DATA

Supplementary Data are available at NAR Online.

## FUNDING

Ministry of Science and Technology of China [2009CB919000, 2011CBA00906, 2012CB316500, 2012DFG32160]; National Natural Science Foundation of China [30971431, 31000365, 61273228, 31100068, 91019016]. Funding for open access charge: Ministry of Science and Technology of China.

*Conflict of interest statement*. None declared.

## Supplementary Material

Supplementary Data
