# Supplementary material for: Integrated omics study delineates the dynamics of lipid droplets in Rhodococcus opacus PD630
Source: Nucleic Acids Res. 2013 Oct 21;42(2):1052–64. doi: 10.1093/nar/gkt932 (PMC3902926; doi:10.1093/nar/gkt932)
Supplement: Supplementary Data [file supp_42_2_1052__index.html]

Integrated omics study delineates the dynamics of lipid droplets in Rhodococcus opacus PD630 — Integrated omics study delineates the dynamics of lipid droplets in Rhodococcus opacus PD630 — Supplementary Data 

# Integrated omics study delineates the dynamics of lipid droplets in *Rhodococcus opacus* PD630

## Supplementary Data

files

**Files in this Data Supplement:**

- Supplementary Data - pdf file
- Supplementary Data - xls file
