# Supplementary material for: Integrated omics study delineates the dynamics of lipid droplets in Rhodococcus opacus PD630
Source: Nucleic Acids Res. 2013 Oct 21;42(2):1052–64. doi: 10.1093/nar/gkt932 (PMC3902926; doi:10.1093/nar/gkt932)
Supplement: Supplementary Data [file supp_gkt932_nar-02216-h-2013-File008.pdf]

Supplementary Figures and Legends

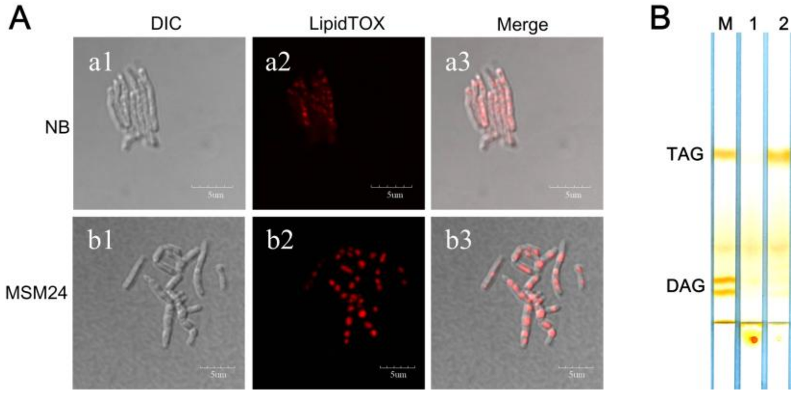

Figure S1 Confocal images of LD and TAG detection of different cultures. (A) Groups a and b, Olympus FV1000 confocal images of *R. opacus* PD630 cells cultivated in nutrient broth (NB) or mineral salt medium (MSM). Cells were stained with LipidTOX. Bars = 5 µm. (B) TLC analysis of total lipids extracted from the same amount of cells, normalized by protein from NB (lane 1) and MSM (lane 2). MSM cells contained significantly more TAG than NB cells. The plate was visualized by iodine vapor. M, marker. TAG, triacylglycerol; DAG, diacylglycerol; MAG, monoglyceride.

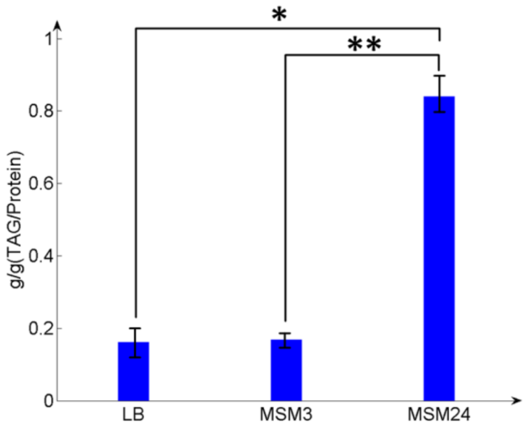

Figure S2 Measurements of TAG contents in the three cultures. TAG content was tested according to protocol of Tissue glyceride assay kit of APPLYGEN. Protein content was tested followed by the instructions of Total Protein Assay Kits of Thermo Scientific. Data are means from three independent experiments; error bars show s.d.; *p <0.01 (LB vs MSM24, 0.00102) and **P < 0.001 (MSM3 vs MSM24, 0.0009), two tailed *t*-test.

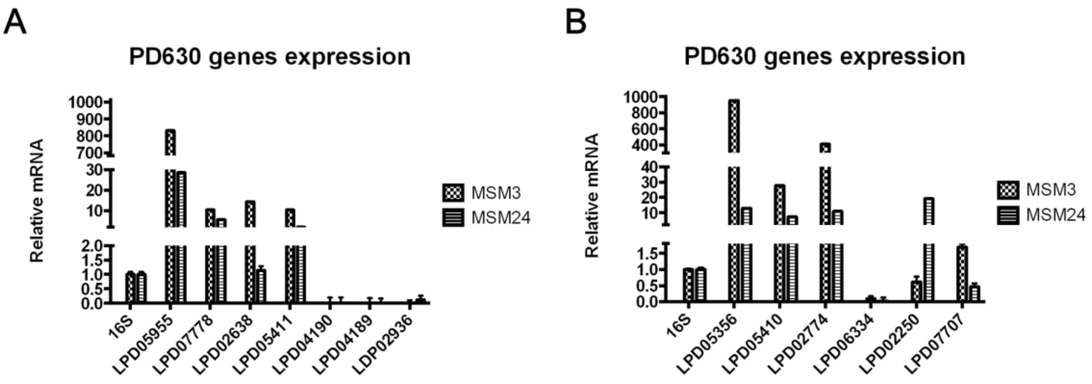

Figure S3 Expression of *R. opacus* PD630 genes in NB, MSM3, and MSM24. Two independent quantitative PCRs (qPCR) were conducted to measure expression of *R. opacus* PD630 genes. Briefly, total RNA from *R. opacus* PD630 was isolated, transcribed into cDNA with M-MLV Reverse Transcriptase, and subjected to qPCR. Relative expression of mRNAs was determined after normalization with 16S levels using the DD-Ct method and compared MSM3, MSM24 with NB, respectively. (A) qPCR of non-LD protein genes in *R. opacus* PD630 from MSM3 and MSM24 against NB. (B) qPCR of LD protein genes in *R. opacus* PD630 from MSM3 and MSM24 against NB.

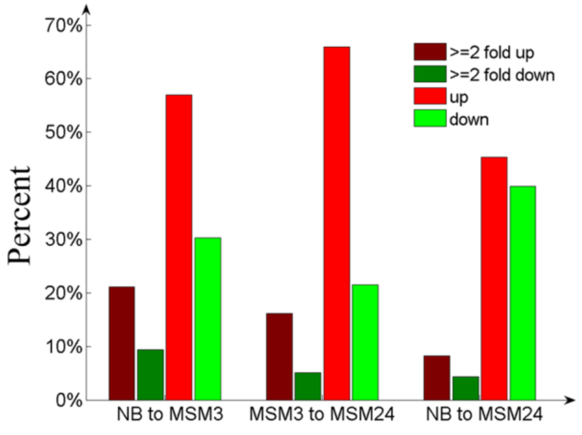

Figure S4 Ratios of differentially expressed genes. Histogram showing the percentage of whole genome genes with expression changes in the three conditions.

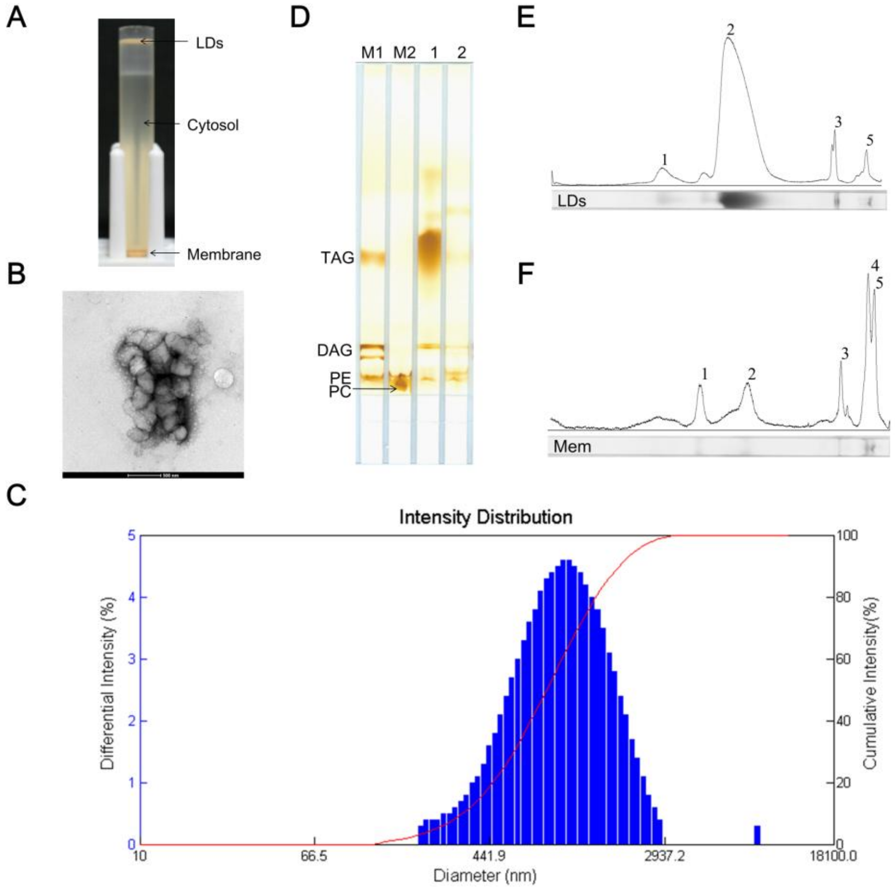

Figure S5 Isolation of LDs from *R. opacus* PD630. (A) Purified LDs from *R. opacus* PD630 were located at the top of the gradient in the SW40 tube. Cytosol was located in the central region and the membrane pellet at the bottom of the tube. (B) EM image of isolated LDs using negative staining. Bar = 500 nm. The sample was loaded onto a carbon-coated, formvar-covered copper grid and then stained for 1 min with 0.5% neutral phosphotungstic acid. The grid was then viewed with a FEI Tecnai 20 (FEI Co., Netherlands) electron microscope. (C) Size distribution of purified LDs. Values were generated with a Delsa Nano C particle analyzer and plotted using Matlab. (D) TLC analysis of total lipids extracted from isolated LDs (lane 1) and the total membrane (lane 2). M1, marker 1, including TAG, DAG, MAG and PE (phosphatidylethanolamine); M2, marker 2, including PE and PC (phosphatidylcholine). (E,F) Semi-quantification of neutral lipid content by grayscale scanning (NIH Image J). Different peak areas indicate the content of each lipid component. 1, unknown neutral lipid; 2, TAG; 3, DAG; 4, Unknown lipid; 5, PE.

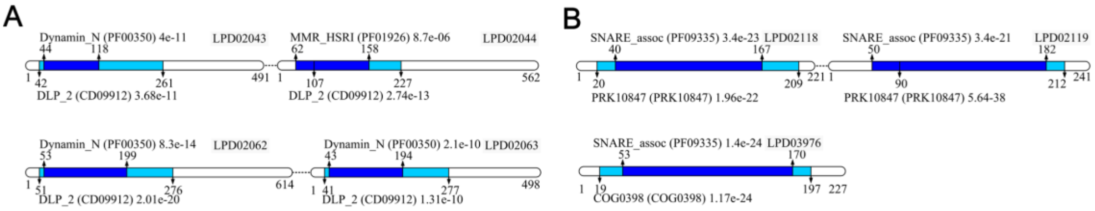

Figure S6 Predicted 4 Dynanin-like proteins and 3 SNARE-like proteins. The 4 Dynanin-like proteins are grouped into 2 operons (LPD02043 and LPD02044; LPD02062 and LPD02063) and 2 SNARE-like proteins (LPD02118 and LPD02119) grouped into an operon. The domains are all predicted by using Pfam and and NCBI CDD database.(A) Dynamin-like protein and its predicted functional domain. (B) SNARE-like protein and its predicted functional domain.

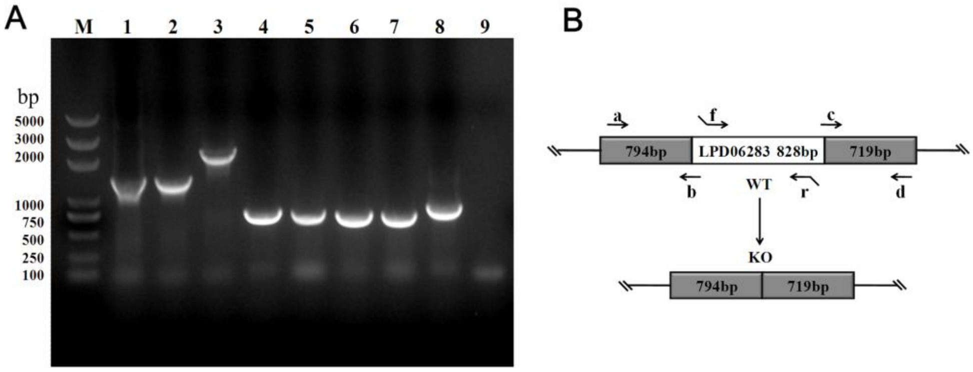

Figure S7 Diagram and confirmation of the LPD06283 gene deletion. (A) PCR confirm of the LPD06283 gene deletion. M, Marker. Lane 1, positive control, PCR fragment of the LPD06283 gene obtained using primers a/d and the knockout pK18mobsacB plasmid as the template; Lane 2, PCR fragment of the LPD06283 gene in the deletion mutant of about 1513 bp, the same as the positive control; Lane 3, negative control, a fragment of about 2344 bp was obtained using *R. opacus* PD630 WT cells as a PCR template; Lane 4-5, upstream flanking sequence of LPD06283 was amplified by primers a and b using WT and deletion mutant genomic DNA as templates; Lane 6-7, downstream flanking sequence of LPD06283 was amplified by primers c and d using WT and deletion mutant genomic DNA as templates. Lane 8-9, the target gene LPD06283 was amplified by primers f and r using WT and deletion mutant genomic DNA as templates. (B) Diagram of the LPD06283 gene deletion showing the used primers and the gene length.

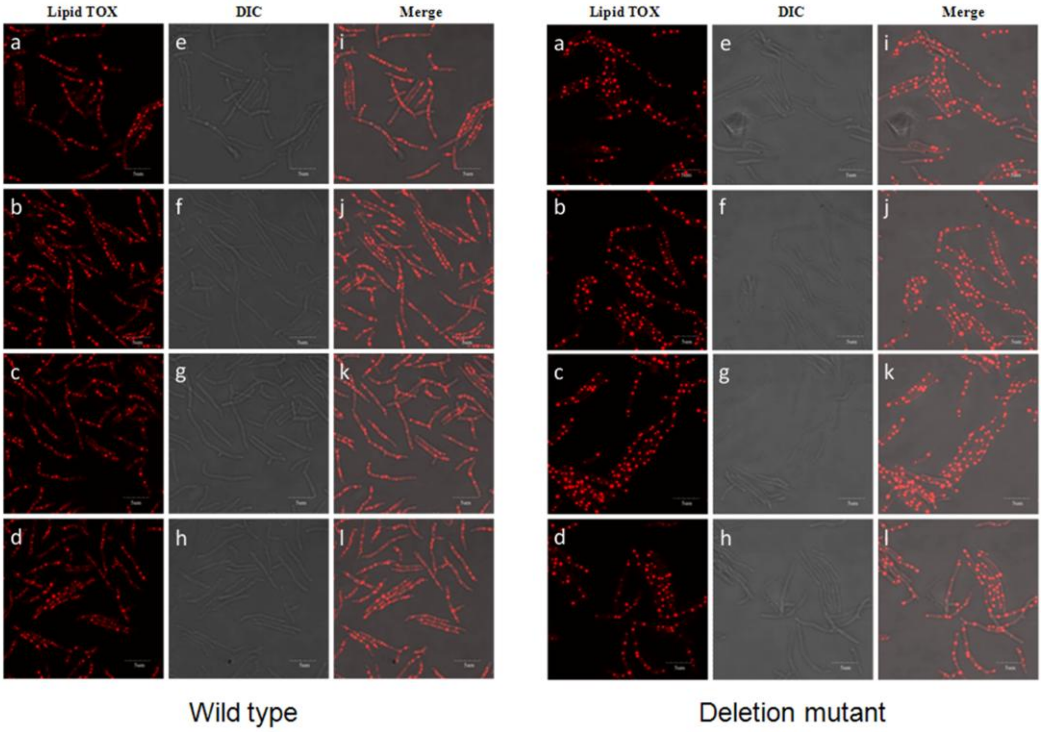

Figure S8 LDs in wild type and LPD06283 deletion mutant. The cells of *R. opacus* PD630 wild type (Wild Type) and LPD06283 deletion mutant (Deletion Mutant) were cultured in NB for 48 h, and transferred to MSM for 12 h, stained by LipidTOX Red, and then examined by confocal microscope. (a-d), fluorescent phase; (e-h), DIC; (i-l), merged (Bars = 5 μm).

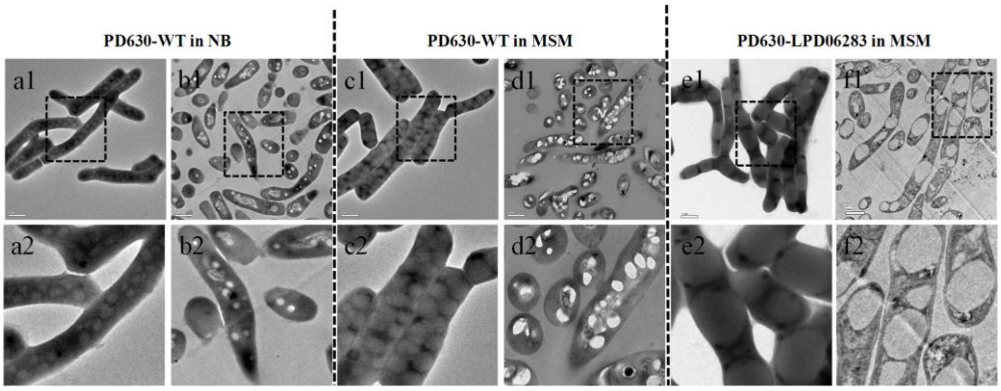

Figure S9 LDs in wild type and the LPD06283 deletion mutant. (a1-b1), *R. opacus* PD630 wild type cells cultured in NB and viewed by EM. (c1-d1), *R. opacus* PD630 wild type cells cultured in MSM and viewed by EM. (e1-f1), Cells of LPD06283 deletion mutant cultured in MSM and viewed by EM. (a2-f2), partially enlarged from A1-F1 respectively. Samples of a1, a2, c1, c2, e1, e2 were prepared following the positive staining method described in Materials and Methods. Samples of b1, b2, d1, d2, f1, f2 were prepared following the ultra thin section method described in Materials and Methods. Bars = 1 μm.
